# The consequences of out-of-hours hip fracture surgery: insights from a retrospective nationwide study

**DOI:** 10.1007/s00068-021-01804-y

**Published:** 2021-10-07

**Authors:** Maximilian Peter Forssten, Ahmad Mohammad Ismail, Tomas Borg, Yang Cao, Per Wretenberg, Gary Alan Bass, Shahin Mohseni

**Affiliations:** 1 Department of Orthopedic Surgery, Orebro University Hospital, 701 85 Orebro, Sweden; 2 School of Medical Sciences, Orebro University, 702 81 Orebro, Sweden; 3 Clinical Epidemiology and Biostatistics, School of Medical Sciences, Orebro University, 701 82 Orebro, Sweden; 4 Division of Traumatology, Emergency Surgery and Surgical Critical Care, University of Pennsylvania, Philadelphia, USA; 5 Division of Trauma and Emergency Surgery, Department of Surgery, Orebro University Hospital, 701 85 Orebro, Sweden

**Keywords:** Hip fracture, Time of day, Internal fixation, Arthroplasty, On hour, Out of hours, Mortality, Surgery

## Abstract

**Purpose:**

The study aimed to investigate the association between out-of-hours surgery and postoperative mortality in hip fracture patients. Furthermore, internal fixation and arthroplasty were compared to determine if a difference could be observed in patients operated with these techniques at different times during the day.

**Methods:**

All patients above 18 of age years in Sweden who underwent hip fracture surgery between 2008 and 2017 were eligible for inclusion. Pathological fractures, non-operatively managed fractures, or cases whose time of surgery was missing were excluded. The cohort was subdivided into on-hour (08:00–17:00) and out-of-hours surgery (17:00–08:00). Poisson regression with adjustments for confounders was used to evaluate the association between out-of-hours surgery and both 30-day and 90-day postoperative mortality.

**Results:**

Out-of-hours surgery was associated with a 5% increase in the risk of both 30-day [adj. IRR (95% CI) 1.05 (1.00–1.10), *p* = 0.040] and 90-day [adj. IRR (95% CI) 1.05 (1.01–1.09), *p* = 0.005] mortality after hip fracture surgery compared to on-hour surgery. There was no statistically significant association between out-of-hours surgery and postoperative mortality among patients who received an internal fixation. Arthroplasties performed out-of-hours were associated with a 13% increase in 30-day postoperative mortality [adj. IRR (95% CI) 1.13 (1.04–1.23), *p* = 0.005] and an 8% increase in 90-day postoperative mortality [adj. IRR (95% CI) 1.08 (1.01–1.15), *p* = 0.022] compared to on-hour surgery.

**Conclusion:**

Out-of-hours surgical intervention is associated with an increase in both 30- and 90-day postoperative mortality among hip fracture patients who received an arthroplasty, but not among patients who underwent internal fixation.

## Introduction

Clinical controversy remains regarding the optimal timing of hip fracture surgery [1–5]. A recent randomized control trial (RCT), the HIP ATTACK study, failed to demonstrate an overall association between expedited surgery (within 6 h of hip fracture diagnosis) and the abrogation of the risk of postoperative complications or death [6]. However, granular analysis suggests specific patient populations, such as those in need of internal fixation of displaced femoral neck fractures may benefit from earlier operative intervention [7–9]. In contrast, those requiring formal arthroplasty may be hypothesized to accrue a more considerable benefit from preoperative optimization [10, 11].

There is great global heterogeneity in unscheduled access to the operating room, limited by philosophy, logistics, and resources. In some health care systems (such as the United Kingdom), out-of-hours surgery is sanctioned only for true threats to life or limb [12, 13], while in others, orthopedic trauma surgical practice patterns, including hip fracture surgery, facilitate and encourage out-of-hours intervention [14]. As the global population ages and this injury pattern becomes more prevalent, an evidence base for the optimal timing of the surgical treatment of hip fractures will become even more relevant [15–17]. This vulnerable, highly comorbid patient population places a heavy burden on health care systems [18–24]. It is, therefore, vital that all avenues are explored to achieve a reduction in mortality, which has hitherto remained relatively constant despite efforts taken to reduce this during the past decades [18, 25]. Previous studies investigating the effect of time-of-day on postoperative mortality have observed varying outcomes. A large study including all orthopedic trauma was able to find an association between out-of-hours surgery and increased postoperative mortality [26]. To date, studies that specifically focused on hip fracture surgery were unable to detect a similar association [27–34]. Interpretation of these studies, however, is limited by relatively small sample populations [27–33].

In this study, we aimed to investigate the association between out-of-hours surgery and postoperative mortality in hip fracture patients, using the largest sample population to date, and to determine if a difference could be observed in patients operated with internal fixation compared to those who received an arthroplasty. We hypothesized that out-of-hours surgery is associated with an increased risk of mortality.

## Patients and methods

The study was approved by the Swedish Ethical Review Authority (ref: 2020-03257) and adhered to the STROBE guidelines and the Declaration of Helsinki [35]. Data were primarily retrieved from Rikshoft, the prospectively collected Swedish National Quality Registry for Hip Fracture Patients [36]. All adult patients (18 years or older) in Sweden who underwent hip fracture surgery between January 1, 2008, and December 31, 2017, were eligible for inclusion. Cases where the fracture was pathological, non-operatively managed, or whose time of surgery was missing were excluded from further analysis. Patient data from Rikshoft was cross-referenced with patients’ preoperative comorbidities and time of death in the Swedish National Board of Health and Welfare’s Patient and Cause of Death Registers. Comorbidities were used to calculate the Charlson Comorbidity Index (CCI) [37].

### Statistical analysis

Cases were divided into two cohorts based on if the surgery was initiated on-hours (08:00–17:00) or out-of-hours (17:00–08:00). The timespans are based on the working hours for the overwhelming majority of healthcare providers in Sweden, where the “on-call” schedule starts at 17:00. Subsequently, patient demographics, clinical characteristics, and crude outcomes were compared between these two shifts using the appropriate statistical tests. Continuous, normally distributed variables were presented as a mean and standard deviation (SD), with the Student’s *t* test being used to evaluate the statistical significance of differences between the groups. Non-normally distributed, continuous variables were presented as a median and interquartile range (IQR); the Mann–Whitney *U* test was employed to determine the statistical significance of differences. Counts and percentages summarize categorical variables, while Pearson’s Chi-squared test was used to test for independence between the groups.

The primary outcome was 30-day postoperative mortality, with 90-day postoperative mortality being the secondary outcome of interest. We applied two time-bound Poisson regression models with robust standard errors to the data (with 30- and 90-day postoperative mortality as the response variable, respectively) to investigate the association between out-of-hours surgery and mortality. Both models were adjusted for age, sex, American Society of Anesthesiologists (ASA) classification, type of fracture, type of surgery, time to surgery, myocardial infarction, congestive heart failure, peripheral vascular disease, cerebrovascular disease, dementia, chronic obstructive pulmonary disease, connective tissue disease, liver disease, diabetes, chronic kidney disease, local cancer, and metastatic carcinoma. These analyses were repeated on a subset of patients who were operated on using internal fixation (pins/screws, pins/screws with side-plate, or intramedullary nail) or arthroplasty (hemiarthroplasty or total hip replacement) since the latter surgical procedures are considered technically more demanding and time-consuming as well as more physiologically traumatic for the patients. Results are presented as incidence rate ratios (IRRs) with 95% confidence intervals (CIs). Statistical significance was defined as a two-sided *p* value less than 0.05. Analyses were performed using the statistical programming language R (R Foundation for Statistical Computing, Vienna, Austria) [38].

## Results

**Fig. 1:**
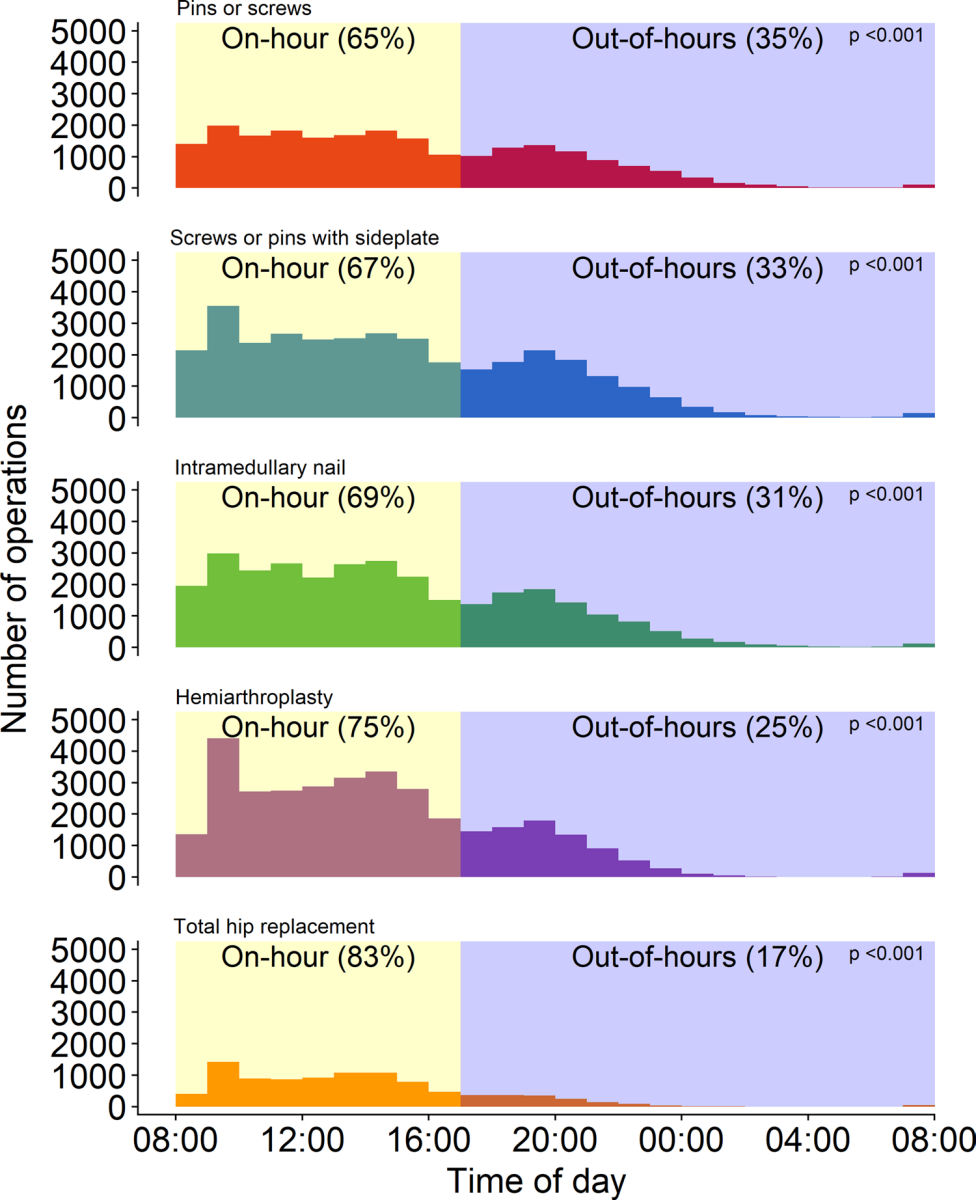
Histogram depicting the distribution of all types of hip fracture surgery based on the time of day. *P* values are calculated using Pearson’s chi-squared test

Based on the inclusion and exclusion criteria, a total of 130,407 hip fracture cases were available for further analysis. A total of 87,253 (67%) received an internal fixation while 43,092 (33%) underwent an arthroplasty. Irrespective of the type of surgery, most patients were operated on-hours (Fig. 1). There was no statistically significant difference in the age or sex of patients in either cohort, as well as no clinically significant difference in the fitness for surgery based on their ASA classification. In general, patients who were operated out-of-hours had a slightly lower comorbidity burden (CCI ≥ 7: 17.7% vs. 19.2%, *p* < 0.001); all comorbidities were less prevalent among patients undergoing out-of-hours surgery, except for hemiplegia, liver disease, and peptic ulcer disease. Patients who were operated out-of-hours were more likely to undergo internal fixation (74.2% vs. 63.9%, *p* < 0.001), and to undergo surgery within 24 h of admission (69.6% vs. 68.5%, *p* < 0.001). This was reflected in an overall shorter length of stay (7 vs. 8 days, *p* < 0.001), but crude 90-day postoperative mortality was slightly higher (13.6% vs. 13.1%, *p* = 0.008) (Table 1).

**Table 1:** Demographics, clinical characteristics, and outcomes of all patients who underwent hip fracture surgery on-hours (08 → 17) and out-of-hours (17 → 08)

	On-hour surgery (*N* = 91,982)	Out-of-hours surgery (*N* = 38,425)	*P* value
Age, mean (SD)	81.9 (± 10)	82.0 (± 10.2)	0.345
Sex, *n* (%)			0.775
Female	62,722 (68.2)	26,168 (68.1)	
Male	29,255 (31.8)	12,252 (31.9)	
Missing	5 (0.0)	5 (0.0)	
ASA classification, *n* (%)			< 0.001
1	4280 (4.7)	2087 (5.4)	
2	32,630 (35.5)	14,010 (36.5)	
3	46,208 (50.2)	18,647 (48.5)	
4	7116 (7.7)	2935 (7.6)	
5	93 (0.1)	36 (0.1)	
Missing	1655 (1.8)	710 (1.8)	
Type of fracture, *n* (%)			< 0.001
Non-displaced cervical (garden 1–2)	11,259 (12.2)	5896 (15.3)	
Displaced cervical (garden 3–4)	36,576 (39.8)	11,984 (31.2)	
Basicervical	2974 (3.2)	1347 (3.5)	
Pertrochanteric (two fragments)	17,063 (18.6)	8621 (22.4)	
Pertrochanteric (multiple fragments)	16,547 (18.0)	7474 (19.5)	
Subtrochanteric	7526 (8.2)	3094 (8.1)	
Missing	37 (0.0)	9 (0.0)	
Type of surgery, *n* (%)			< 0.001
Pins or screws	14,644 (15.9)	7834 (20.4)	
Screws or pins with side-plate	22,690 (24.7)	11,102 (28.9)	
Intramedullary nail	21,411 (23.3)	9572 (24.9)	
Hemiarthroplasty	25,253 (27.5)	8214 (21.4)	
Total hip replacement	7941 (8.6)	1684 (4.4)	
Missing	43 (0.0)	19 (0.0)	
Time to surgery, *n* (%)			< 0.001
< 24 h	62,975 (68.5)	26,744 (69.6)	
≥ 24 h	29,007 (31.5)	11,681 (30.4)	
Charlson Comorbidity Index, *n* (%)			< 0.001
≤ 4	39,419 (42.9)	17,810 (46.4)	
5–6	34,896 (37.9)	13,810 (35.9)	
≥ 7	17,667 (19.2)	6805 (17.7)	
Myocardial infarction, *n* (%)	5612 (6.1)	2214 (5.8)	0.019
Congestive heart failure, *n* (%)	14,886 (16.2)	5690 (14.8)	< 0.001
Hypertension, *n* (%)	36,764 (40.0)	13,828 (36.0)	< 0.001
Arrhythmia, *n* (%)	17,650 (19.2)	6780 (17.6)	< 0.001
Peripheral vascular disease, *n* (%)	4149 (4.5)	1562 (4.1)	< 0.001
Cerebrovascular disease, *n* (%)	16,340 (17.8)	6418 (16.7)	< 0.001
Dementia, *n* (%)	19,322 (21.0)	7227 (18.8)	< 0.001
COPD, *n* (%)	11,035 (12.0)	4172 (10.9)	< 0.001
Connective tissue disease, *n* (%)	4558 (5.0)	1748 (4.5)	0.002
Peptic ulcer disease, *n* (%)	3012 (3.3)	1218 (3.2)	0.339
Liver disease, *n* (%)	968 (1.1)	363 (0.9)	0.083
Diabetes, *n* (%)	13,745 (14.9)	5493 (14.3)	0.003
Hemiplegia, *n* (%)	2062 (2.2)	796 (2.1)	0.058
Chronic kidney disease, *n* (%)	4946 (5.4)	1848 (4.8)	< 0.001
Local tumor, *n* (%)	10,113 (11.0)	4044 (10.5)	0.013
Metastatic carcinoma, *n* (%)	2094 (2.3)	805 (2.1)	0.045
Length of stay, median [IQR]	8.0 [5.0–12]	7.0 [4.0–11]	< 0.001
Missing, *n* (%)	697 (0.8)	273 (0.7)	
30-Day mortality, *n* (%)	6877 (7.5)	2990 (7.8)	0.059
90-Day mortality, *n* (%)	12,022 (13.1)	5234 (13.6)	0.008

*ASA* American Society of Anesthesiologists, *COPD* chronic obstructive pulmonary disease

When comparing the cohorts in the subset of patients operated with internal fixation, differences between the cohorts were relatively unchanged compared to the entire patient population. The primary change was that there was no statistically significant difference in the proportion of patients who underwent surgery within 24 h (72.0% vs. 71.4%, *p* = 0.060). Length of stay was still shorter among patients who were operated out-of-hours (7 vs. 8 days, *p* < 0.001), but there was no statistically significant difference in crude 30- or 90-day postoperative mortality (Table 2).

**Table 2:** Demographics, clinical characteristics, and outcomes of patients who underwent hip fracture surgery with internal fixation on-hours (08 → 17) and out-of-hours (17 → 08)

	On-hour surgery (*N* = 58,745)	Out-of-hours surgery (*N* = 28,508)	*P* value
Age, mean (SD)	81.7 (± 10.7)	81.5 (± 10.9)	0.015
Sex, *n* (%)			0.170
Female	39,997 (68.1)	19,276 (67.6)	
Male	18,744 (31.9)	9228 (32.4)	
Missing	4 (0.0)	4 (0.0)	
ASA classification, *n* (%)			< 0.001
1	2918 (5.0)	1690 (5.9)	
2	20,169 (34.3)	10,331 (36.2)	
3	29,455 (50.1)	13,669 (47.9)	
4	5021 (8.5)	2251 (7.9)	
5	75 (0.1)	26 (0.1)	
Missing	1107 (1.9)	541 (1.9)	
Type of fracture, *n* (%)			< 0.001
Non-displaced cervical (garden 1–2)	10,128 (17.2)	5535 (19.4)	
Displaced cervical (garden 3–4)	5413 (9.2)	2696 (9.5)	
Basicervical	2425 (4.1)	1188 (4.2)	
Pertrochanteric (two fragments)	16,912 (28.8)	8567 (30.1)	
Pertrochanteric (multiple fragments)	16,450 (28.0)	7448 (26.1)	
Subtrochanteric	7402 (12.6)	3067 (10.8)	
Missing	15 (0.0)	7 (0.0)	
Type of surgery, *n* (%)			< 0.001
Pins or screws	14,644 (24.9)	7834 (27.5)	
Screws or pins with side-plate	22,690 (38.6)	11,102 (38.9)	
Intramedullary nail	21,411 (36.4)	9572 (33.6)	
Time to surgery, *n* (%)			0.060
< 24 h	41,929 (71.4)	20,523 (72.0)	
≥ 24 h	16,816 (28.6)	7985 (28.0)	
Charlson Comorbidity Index, *n* (%)			< 0.001
≤ 4	25,071 (42.7)	13,483 (47.3)	
5–6	22,312 (38.0)	10,012 (35.1)	
≥ 7	11,362 (19.3)	5013 (17.6)	
Myocardial infarction, *n* (%)	3721 (6.3)	1607 (5.6)	< 0.001
Congestive heart failure, *n* (%)	9912 (16.9)	4216 (14.8)	< 0.001
Hypertension, *n* (%)	23,085 (39.3)	10,052 (35.3)	< 0.001
Arrhythmia, *n* (%)	11,158 (19.0)	4933 (17.3)	< 0.001
Peripheral vascular disease, *n* (%)	2715 (4.6)	1173 (4.1)	< 0.001
Cerebrovascular disease, *n* (%)	10,484 (17.8)	4700 (16.5)	< 0.001
Dementia, *n* (%)	12,423 (21.1)	5201 (18.2)	< 0.001
COPD, *n* (%)	7335 (12.5)	3156 (11.1)	< 0.001
Connective tissue disease, *n* (%)	2698 (4.6)	1261 (4.4)	0.267
Peptic ulcer disease, *n* (%)	1992 (3.4)	904 (3.2)	0.093
Liver disease, *n* (%)	662 (1.1)	275 (1.0)	0.032
Diabetes, *n* (%)	8870 (15.1)	4080 (14.3)	0.002
Hemiplegia, *n* (%)	1323 (2.3)	602 (2.1)	0.194
Chronic kidney disease, *n* (%)	3146 (5.4)	1355 (4.8)	< 0.001
Local tumor, *n* (%)	6343 (10.8)	3013 (10.6)	0.312
Metastatic carcinoma, *n* (%)	1282 (2.2)	571 (2.0)	0.089
Length of stay, median [IQR]	8.0 [5.0–12]	7.0 [4.0–11]	< 0.001
Missing, *n* (%)	437 (0.7)	195 (0.7)	
30-Day mortality, *n* (%)	4493 (7.6)	2132 (7.5)	0.382
90-Day mortality, *n* (%)	8020 (13.7)	3860 (13.5)	0.658

*ASA* American Society of Anesthesiologists, *COPD* chronic obstructive pulmonary disease

Patients undergoing arthroplasty exhibited similar demographics to the entire patient population. There was no clinically significant difference in age and no statistically significant difference in sex, type of fracture, time to surgery, fitness for surgery according to ASA classification, or comorbidity burden based on CCI. The only differences in comorbidity prevalence were seen in hypertension, arrhythmia, chronic obstructive pulmonary disease, connective tissue disease, and local tumors, all of which were less prevalent among patients who underwent surgery out-of-hours. Both crude 30-day (8.6% vs. 7.2%, *p* < 0.001) and 90-day (13.9% vs. 12.0%, *p* < 0.001) postoperative mortality was higher among patients who were operated out-of-hours (Table 3).

**Table 3:** Demographics, clinical characteristics, and outcomes of patients who underwent hip fracture surgery with arthroplasty on-hours (08 → 17) and out-of-hours (17 → 08)

	On-hour surgery (*N* = 33,194)	Out-of-hours surgery (*N* = 9898)	*P* value
Age, mean (SD)	82.3 (± 8.4)	83.4 (± 7.8)	< 0.001
Sex, *n* (%)			0.033
Female	22,692 (68.4)	6879 (69.5)	
Male	10,501 (31.6)	3018 (30.5)	
Missing	1 (0.0)	1 (0.0)	
ASA classification, *n* (%)			0.128
1	1360 (4.1)	396 (4.0)	
2	12,444 (37.5)	3672 (37.1)	
3	16,740 (50.4)	4972 (50.2)	
4	2090 (6.3)	681 (6.9)	
5	18 (0.1)	10 (0.1)	
Missing	542 (1.6)	167 (1.7)	
Type of fracture, *n* (%)			0.535
Non-displaced cervical (garden 1–2)	1127 (3.4)	357 (3.6)	
Displaced cervical (garden 3–4)	31,150 (93.8)	9284 (93.8)	
Basicervical	548 (1.7)	159 (1.6)	
Pertrochanteric (two fragments)	142 (0.4)	47 (0.5)	
Pertrochanteric (multiple fragments)	90 (0.3)	23 (0.2)	
Subtrochanteric	120 (0.4)	26 (0.3)	
Missing	17 (0.1)	2 (0.0)	
Type of surgery, *n* (%)			< 0.001
Hemiarthroplasty	25,253 (76.1)	8214 (83.0)	
Total hip replacement	7941 (23.9)	1684 (17.0)	
Time to surgery, *n* (%)			0.278
< 24 h	21,017 (63.3)	6207 (62.7)	
≥ 24 h	12,177 (36.7)	3691 (37.3)	
Charlson Comorbidity Index, *n* (%)			0.141
≤ 4	14,328 (43.2)	4320 (43.6)	
5–6	12,569 (37.9)	3788 (38.3)	
≥ 7	6297 (19.0)	1790 (18.1)	
Myocardial infarction, *n* (%)	1890 (5.7)	606 (6.1)	0.115
Congestive heart failure, *n* (%)	4961 (14.9)	1472 (14.9)	0.869
Hypertension, *n* (%)	13,666 (41.2)	3772 (38.1)	< 0.001
Arrhythmia, *n* (%)	6489 (19.5)	1844 (18.6)	0.044
Peripheral vascular disease, *n* (%)	1434 (4.3)	388 (3.9)	0.088
Cerebrovascular disease, *n* (%)	5853 (17.6)	1716 (17.3)	0.507
Dementia, *n* (%)	6893 (20.8)	2022 (20.4)	0.476
COPD, *n* (%)	3692 (11.1)	1013 (10.2)	0.014
Connective tissue disease, *n* (%)	1859 (5.6)	486 (4.9)	0.008
Peptic ulcer disease, *n* (%)	1015 (3.1)	313 (3.2)	0.621
Liver disease, *n* (%)	305 (0.9)	88 (0.9)	0.831
Diabetes, *n* (%)	4873 (14.7)	1410 (14.2)	0.289
Hemiplegia, *n* (%)	739 (2.2)	194 (2.0)	0.119
Chronic kidney disease, *n* (%)	1797 (5.4)	493 (5.0)	0.097
Local tumor, *n* (%)	3767 (11.3)	1030 (10.4)	0.009
Metastatic carcinoma, *n* (%)	810 (2.4)	234 (2.4)	0.693
Length of stay, median [IQR]	8.0 [5.0–12]	8.0 [5.0–12]	< 0.001
Missing, *n* (%)	251 (0.8)	75 (0.8)	
30-Day mortality, *n* (%)	2378 (7.2)	856 (8.6)	< 0.001
90-Day mortality, *n* (%)	3995 (12.0)	1372 (13.9)	< 0.001

*ASA* American Society of Anesthesiologists, *COPD* chronic obstructive pulmonary disease

After adjusting for potential confounders, out-of-hours surgery was associated with a 5% increase in the risk of both 30-day [adj. IRR (95% CI) 1.05 (1.00–1.10), *p* = 0.040] and 90-day [adj. IRR (95% CI) 1.05 (1.01–1.09), *p* = 0.005] mortality after hip fracture surgery compared to on-hour surgery. There was no statistically significant association between out-of-hours surgery and postoperative mortality among patients who received an internal fixation. Arthroplasties performed out-of-hours were associated with a 13% increase in 30-day postoperative mortality [adj. IRR (95% CI) 1.13 (1.04–1.23), *p* = 0.005] and an 8% increase in 90-day postoperative mortality [adj. IRR (95% CI) 1.08 (1.01–1.15), *p* = 0.022] compared to on-hour surgery (Table 4).

**Table 4:** Incidence rate ratios for 30- and 90-day mortality after hip fracture surgery

	30-Day IRR (95% CI)	*P* value	90-Day IRR (95% CI)	*P* value
All patients				
On-hour surgery	Ref		Ref	
Out-of-hours surgery	1.05 (1.00–1.10)	0.040	1.05 (1.01–1.09)	0.005
Internal fixation				
On-hour surgery	Ref		Ref	
Out-of-hours surgery	1.02 (0.97–1.08)	0.479	1.04 (1.00–1.08)	0.062
Arthroplasty				
On-hour surgery	Ref		Ref	
Out-of-hours surgery	1.13 (1.04–1.23)	0.005	1.08 (1.01–1.15)	0.022

Poisson regression models with robust standard errors were employed. All models are adjusted for age, sex, ASA classification, type of fracture, type of surgery, time to surgery, myocardial infarction, congestive heart failure, peripheral vascular disease, cerebrovascular disease, dementia, chronic obstructive pulmonary disease, connective tissue disease, liver disease, diabetes, chronic kidney disease, local cancer, and metastatic carcinoma. Missing values were managed using multiple imputation by chained equations

*IRR* incidence rate ratio, *ASA* American Society of Anesthesiologists

## Discussion

This is the most extensive study to investigate the association between out-of-hours surgery and postoperative mortality in hip fracture patients and the first that compares outcomes following emergency internal fixation and arthroplasty. Poisson regression analysis initially indicated that out-of-hours surgery (17:00–08:00) was associated with a 5% increase in both 30- and 90-day mortality in all hip fracture patients. However, subgroup analyses indicated that there was no association present among patients who received an internal fixation. On the other hand, arthroplasty was associated with a 13% increase in 30-day postoperative mortality and an 8% increase in 90-day postoperative mortality.

The effect of out-of-hours procedures has been previously investigated in orthopedic surgery, with conflicting results [26–34]. Orthopedic trauma surgery performed during the afternoon and at night is associated with an increase in mortality [26]. However, none of the studies that specifically focused on hip fracture surgery could detect a statistically significant association between out-of-hours surgery and postoperative mortality [27–34]. A straightforward explanation for this could be a lack of statistical power, as most of these studies were only 1/100^th^ the size of the current study [27–33]. Furthermore, just two of the hip fracture studies appear to have performed adjustments for significant covariates such as comorbidities and ASA classification in their analyses of out-of-hours surgery [33, 34], which increases the risk that their results are affected by residual confounding [27–32].

Any association between out-of-hours operating and increased postoperative mortality, while much studied, is likely to be multifactorial and not simply a sequel of surgeon fatigue. The current literature has yet to provide conclusive evidence that fatigue results in a decline in *surgical* performance [39–44]. Moreover, as can be seen in Fig. 1, the majority of out-of-hours operations are performed before midnight, with the peak lying between 19:00 and 20:00; this skewing of the procedures to the early portion of the out-of-hours shift minimizes the risk of surgeon fatigue playing a significant role in the observed increase in postoperative mortality out-of-hours. Another explanation that might be proposed is surgical experience. In general, the out-of-hours tend to be staffed by senior residents or junior specialists, with less access to more experienced surgeons than regular work hours; however, this also seems unlikely to be the cause of the discrepancy. According to the literature, except in rare circumstances, hip fracture surgery itself does not kill patients; they die due to cardiovascular, respiratory, or cerebrovascular events, among other causes [21, 45–48]. At most, these events can be attributable to the strain induced by the anesthesia, but even the time spent under anesthesia does not appear to be increased out-of-hours [49].

The effect of the type of fracture being operated on also bears consideration, as arthroplasty is primarily performed on displaced cervical hip fractures, which are intracapsular fractures. As it stands, there is no consensus regarding the effect of intra- versus extracapsular fractures on postoperative mortality [50–54]. However, among the studies that observed a difference, *extracapsular* fractures were the type of fracture that was associated with an increased risk of mortality [50–52]. Consequently, this makes it unlikely that the type of fracture can explain the difference observed between internal fixation and arthroplasty.

A more likely cause, in the authors’ opinion, could be the resources available out-of-hours in the hospital setting. During the day, a patient’s vitals may be controlled several times, and the patient will be checked upon many times more. Out-of-hours, these kinds of controls are significantly reduced, both due to a reduction in staff on the wards and to allow patients to recuperate after surgery. This makes it more challenging to both discover complications as well as detect changes in the patient’s status which would allow surgeons to preempt the development of complications. For example, if a surgical wound starts bleeding during the night, it might go unnoticed until the surgeon rounds the patient the following morning, when the patient already has developed anemia. This could also explain why only arthroplasty was associated with increased postoperative mortality if performed out-of-hours. Arthroplasties are generally more complex and time-consuming procedures with larger blood losses, compared to internal fixation procedures, which might increase the risk of early acute postoperative complications [55].

This study has several significant strengths, such as the large longitudinal sample size, including 10 consecutive years of data from the Swedish National Quality Registry for Hip Fracture Patients, with a high case coverage between 80 and 90% [56], as well as the ability to adjust for a multitude of confounding factors. Nevertheless, the usual limitations of a retrospective administrative dataset apply. We were therefore unable adjust for the type of anesthesia used or other intraoperative variables. We also did not have any access to data regarding the duration of the surgical procedures, the rationale for patient-level decisions regarding operative timing, or the individual surgeon’s experience; however, these are unlikely significant cofounders [21, 45–49].

## Conclusion

Out-of-hours surgical intervention is associated with an increase in both 30- and 90-day postoperative mortality among hip fracture patients who received an arthroplasty, while no such association could be detected among patients who underwent internal fixation. Accordingly, procedures involving arthroplasty should likely be avoided out-of-hours, while internal fixation can be considered and performed around the clock if not contraindicated by patient characteristics and when adequate resources exist.

## Data Availability

All data are available for retrieval upon reasonable request.

## Code availability

Codes are available for retrieval upon reasonable request.
